# Strengthening quality of care in partnership with long-term care facilities: Protocol of the Swiss National Implementation Programme NIP-Q-UPGRADE

**DOI:** 10.1177/00469580251328101

**Published:** 2025-05-22

**Authors:** Nereide A. Curreri, Laurie Corna, Emmanuelle Poncin, Bastiaan Van Grootven, Jianan Huang, Magda Osinska, Serena Sibilio, Lisa Kästner, Simon Thuillard, Lucie Vittoz, Sonja Baumann, Brigitte Benkert, Angelika Rüttimann, Anna Brambilla, Gabriela Cafaro, Nathalie Wellens, Franziska Zúñiga

**Affiliations:** 1 Applied University of Sciences and Arts of Southern Switzerland, (SUPSI), Manno, Switzerland; 2 University of Applied Sciences Western Switzerland, Lausanne, Switzerland; 3 University of Basel, Switzerland

**Keywords:** long term care, older adults, quality development, quality indicators, quality of care

## Abstract

Quality improvement is essential in long-term care for older adults. Reporting medical quality indicators (MQI) is commonplace, but the impact on care quality improvement remains uncertain. This paper presents the Swiss National Implementation Programme - Strengthening Quality of Care in Partnership with Residential Long-Term Care Facilities (LTCF) for Older People (NIP-Q-UPGRADE), that aims to develop quality in Swiss long-term care facilities (LTCFs) by (1) strengthening robustness of MQI data, (2) supporting LTCFs in data-driven quality improvement, (3) introducing further quality indicators. The protocol for implementing the programme is outlined by work package and specific sub-aims. NIP-Q-UPGRADE is grounded in implementation science principles, using EPIS (exploration, preparation, implementation, sustainment) as a process framework and the Consolidated Framework for Implementation Research (CFIR) for its contextual analyses, and it has a strong participatory approach. Sub-studies focus on understanding current context, leveraging expertise, developing and piloting actionable intervention bundles with corresponding strategies, and preparing a national scale-up. Methodologies include literature reviews, ethnographic research, international case studies, intervention mapping, online-surveys, participatory workshops as well as pragmatic trials. At the end of NIP-Q-UPGRADE, we expect to have intervention bundles ready to improve data quality and foster data-driven quality improvement in LTCFs and to have the field prepared with corresponding implementation strategies so that national and regional LTC organizations can plan and monitor the scale-up. NIP-Q-UPGRADE will implement strategies and inform policies for sustainable, data-driven quality development. Results will inform national quality improvement implementation applicable to global LTC policies and practices.

**Highlights**

● The NIP-Q-UPGRADE program uses implementation science frameworks (EPIS, CFIR) and participatory methods to develop and sustain quality improvements in Swiss long-term care facilities.

● The program includes contextual analysis, intervention design, and pilot testing using diverse methodologies such as ethnographic research, international case studies, and pragmatic trials.

● The project aims to deliver actionable intervention bundles and implementation strategies for national scale-up, informing both Swiss and international long-term care policies.

## Introduction

Ensuring quality of care within the long-term care (LTC) sector has become a policy priority in most countries. This is driven by factors such as the growing demand for LTC due to population ageing, rising care costs, and the recognition of the state’s responsibility to protect older adults with care needs.^1^ Moreover, care recipients increasingly expect to have a voice regarding the care they receive.^1,2^ Assessing quality is also essential for maintaining efficiency, securing appropriate funding for the sector and ensuring its sustainability.^3^

### Quality of Care in LTCFs

The World Health Organisation (WHO) defines it as care that is effective, safe, and person-centred, while other definitions emphasize desired health outcomes and overall efficiency.^4^ There is little consensus regarding the definition of quality within the LTC sector or the specific indicators to assess it.^5,6^ Defining quality in residential LTC is complex, as this setting provides a combination of acute, post-acute, chronic, palliative and social care to residents with increasingly complex care needs, where functional decline is expected over time. A common approach is to apply broad quality guidelines used in health and social care and adapt minimum standards typically related to structural criteria, such as the configuration of space^2,6^ or to identify problems with care (or the lack thereof) by measuring the prevalence of issues like pressure ulcers or falls.^1^ Less commonly assessed aspects of quality include the resident’s perspective on their quality of life, preferences, and satisfaction with their social environment.

While quality standards and assurance are deemed necessary pillars of LTC, the systematic methods of measuring and monitoring quality remain a topic of debate.^5,7^ Even though medical and clinical quality indicators are widespread, fewer focus on patient reported or client driven quality measures that explore subjective experiences, outcomes, and satisfaction with services.^8^

Understanding quality in care homes encompasses both the subjective experience of well-being and the objective components of ‘a good life’.^9^ Kane and Cutler^10^ (2015) offer a 3-pronged framework for assessing long-term care services focussing on 3 key domains of quality: environment (private and shared spaces), philosophy (control and choice), and service capacity (routine and specialized services). Additionally, long-term care quality is shaped by factors such as education and training for care home managers, residents’ rights and responsibilities, and criteria related to space, staffing, and service capacity.^6,10^ A literature review on the meaning of quality in long-term care further identifies 9 themes rooted in quality-of-life and well-being concepts, expanding the focus beyond clinical aspects to emphasize person-centred care through needs, choices, and personal goals^11^. A summary of these aspects is found in the conceptual model of quality of care in nursing homes of the National Academies of Science, Engineering and Medicine^12^ post-pandemic report (2022), which stresses the resident as the central focus, and encompasses the key categories of care, communication/collaboration, empowered staff and environment.

The data-driven development of quality care, leveraging routinely collected data to guide improvements, is a prominent topic in residential long-term care.^12-14^ A recent survey of publicly reported quality indicators in residential long-term care showed the lack of information about their methodological quality, including their accuracy in reflecting the residents’ situation.^14^ However, data-driven quality development relies on robust quality indicator data and feedback reports that clearly identify issues and changes needed.^12^ A 2019 Swiss survey highlights concern about the clarity of the measurement items and the practical use of the quality indicators for data-driven quality improvement.^15^

### The Swiss Context

The federalist system in Switzerland provides a national legal framework for health and long-term care, but both are governed at the cantonal level, resulting in inconsistency in quality monitoring across cantons.^16^ Beyond federal requirements, cantons can independently mandate quality monitoring systems and improvement initiatives that may include training and monitoring for certain periods of time on specific themes such as pressure ulcers or falls. Depending on the canton, care providers may be monitored more closely with varying quality criteria.^16^

The federal legal requirement for all LTCFs to collect and transmit data on medical quality indicators was integrated into the Swiss Federal Law on Compulsory Health Care (LAMal article 59*a*) in 1994.^17^ As of 2019, all facilities transmit annual standardized resident outcome data on 6 national medical quality indicators (MQIs) in 4 thematic areas to the Federal Statistical Office: polypharmacy, pain (resident-reported and observer-rated), physical restraints (bedrails and seating that restricts liberty/trunk fixation) and malnutrition (weight loss).^18^ Additional indicators on 3 themes are currently being prepared for introduction: pressure ulcers, advanced care planning, and medication review. While the Swiss Federal Law mandates the collection of MQI data and provides public reporting, there is variation in the processes of resident assessment between facilities. Swiss LTC are obliged to work with a resident assessment instrument for reimbursement reasons. For historical reasons and lacking a legal basis for a unified assessment instrument, 3 different resident assessment instruments are in use in Switzerland, one of them interRAI. Accordingly, the MQI selected are related to the quality indicators by interRAI, but do not fully align (eg, in Switzerland, polypharmacy is measured as 9 and more active ingredients and not 9 and more medications). In February of 2024, for the first time, the data on the MQIs from all 1300 LTCFs in Switzerland from 2021 were made public by the Federal Office of Public Health.

Currently, the existing measurement themes focus exclusively on outcome indicators, underscoring the need to consider additional process or structural indicators for a more holistic assessment of care quality. The introduction of new quality indicators needs preparation, since their purpose, relevance, and scientific evidence needs to be considered in collaboration with stakeholders.^19^

At this point in time, an assessment of the quality of the data, its use in practice for guiding data-driven quality improvement, and the preparation of the field for the introduction of additional quality indicators is needed. For this reason, the Federal Quality Commission has mandated the National Implementation Programme – Strengthening Quality of Care in Partnership with Residential Long-Term Care Facilities for Older People (NIP-Q-UPGRADE). NIP-Q-UPGRADE is led by ARTISET, the national federation of service providers for people in need of assistance and its industry association, CURAVIVA, and senesuisse, a national association of private LTCFs. NIP-Q-UPGRADE is implemented in 3 Swiss language regions (German-, French-, and Italian-speaking regions), by academic partners from a three-university consortium representing each linguistic region: the Institute for Nursing Science (INS) at the University of Basel, the Institut et Haute École La Source in Lausanne (La Source), and the Competence Centre on Ageing at the University of Applied Sciences & Arts of Southern Switzerland (SUPSI).

### Objectives

The overarching goal of NIP-Q-UPGRADE is to support LTCFs in continuous improvement of quality of care. The programme is divided into 3 work packages to address its main objectives: (1) to strengthen the quality and robustness of MQI data collected; (2) to support LTCFs in further developing and implementing data-driven care quality improvement; and (3) to prepare the field for the introduction of additional MQIs.

## Methods

### Programme Design

NIP-Q-UPGRADE is framed by an implementation science approach, using a range of methodologies to move from pre-implementation to implementation and sustainment of the main theme in each work package. This facilitates the adoption and integration of evidence-based interventions to improve health and social care settings such as LTCFs for older adults.^20^ Specifically, 4 frameworks, described below, guide the design of the programme and the build-up of the individual studies.

### Guiding Frameworks

First, the EPIS framework is applied across the overall programme, from the understanding of the implementation context to preparing, guiding and sustaining the implementation itself. The EPIS framework serves as a process model for implementation with 4 phases: Exploration, Preparation, Implementation, Sustainment.^21^ These 4 phases guide especially work package 1 and 2, each starting with the exploration of the literature and context to understand the issues at hand and possible solutions, followed by the preparation phase with the development and contextualizing of fitting interventions. In the implementation phase, pilot studies and local implementation help to understand how to best support the intervention before scaling up. We follow a participatory approach throughout, by, for example using ethnography and co-participatory workshops to gather contextual data and develop evidence-based interventions. Through the EPIS framework, the work packages were subdivided into steps or sub aims.

Second, the Consolidated Framework for Implementation Research (CFIR) guides us in the analyses of the context in which interventions bundles are to be implemented. CFIR contains 5 domains with associated constructs (characteristics of the intervention, external setting, internal setting, characteristics of the persons involved, process of implementation). The 5 domains allow for a structured understanding of the context. When developing the intervention, the insights from the contextual analysis help to differentiate core elements from contextually adaptable peripheral elements of an intervention and to define implementation strategies appropriate to the context.^22^ Through a participatory process, the contextual determinants identified through CFIR will be considered when developing feasible and acceptable implementation strategies with stakeholders.

Third, the Interactive Systems Framework (ISF) is used throughout the programme to orient the level at which measures and implementations strategies should be introduced. It is used to describe the different roles and relationships of, and within, all systems of the implementation of interventions in LTCFs: the synthesis and translation system, the support system (eg, cantonal associations, educational institutions), and the delivery system (ie, the LTCF and their staff).^23^

Finally, the Phases of Scale-up framework will guide the scale-up of the intervention bundles developed, focussing on the implementation of interventions to strengthen quality in Swiss LTC facilities.^24^ The framework outlines 4 sequential phases that guide scale-up, including: (1) understanding the context and preparing the field where the intervention can be introduced and tested; (2) the development of a scalable unit; (3) testing the intervention in various settings that characterize the setting of the full-scale up; and (4) enabling widespread adoption of the intervention in the desire setting. Particular attention is devoted to the activities necessary to being able to fully scale an intervention, the prerequisite mechanisms that facilitate intervention adoption and the support systems that need to be in place.^24^

Additional details on how each framework is applied will be offered in future publications of individual sub aim studies.

Given the importance of involving stakeholders in all phases of the programme, including opportunities for co-creation, we involve them at all levels. We include representatives from federal and cantonal offices, national and international experts, electronic health record software companies, and resident assessment instrument providers, cantonal LTCF associations, professional organizations (eg, nurses, geriatricians, pharmacists), educational institutions and those who are directly and indirectly involved in care, such as LTCF staff, residents, and their families.^25^ NIP-Q-UPGRADE will be completed in close collaboration with the national LTCF associations to assure they can fully identify with the programme and are ready to take over the responsibility for going to full scale once NIP-Q-UPGRADE is complete.

### Work Packages – Overview

NIP-Q-UPGRADE is structured into 3 work packages (WPs), each with several sub-aims, that focus on specific objectives and aligned methodologies (Table 1). The WPs address aspects of improving data quality (WP1), implementing quality development (WP2), and introducing new national MQIs (WP3).

**Table 1.** Overview of the 3 Work Packages in NIP-Q-UPGRADE.

Work packages and general sub-aims
**Work Package 1: Improvement of data quality**
*Objective: To investigate the current quality of MQI data, develop, test and prepare the scale-up of interventions for improvement*
1.1 Review literature on determinants of data quality, interventions to enhance data quality, communication strategies to enhance MQI data interpretation, implementation strategies for scale-up in LTCFs
1.2 Define criteria for data quality
1.3 (combined with 3.3) Assess current practices of data collection, documentation, determinants of MQI data quality
1.4 Assess current quality of data in Swiss LTCFs
1.6 Assess needs to optimize MQI data communication; develop measures for LTCFs, software providers, federal offices.
1.5 Analyse, improve data quality problems at system level
1.7/1.8 Develop pilot-test interventions to improve data quality
1.9 Evaluate interventions to improve data quality
1.10 Perform regional scale-up, provide recommendations for national scale-up
**Work Package 2: Fostering data-driven quality improvement**
**Objective: To develop and implement data-driven quality improvement targeting Swiss MQI themes in LTCFs**
2.1 Review literature on interventions to improve MQI themes, evaluating scale- up programmes
2.2 Identify lessons learned from international data-driven policy programmes in quality improvement
2.3 Assess current quality improvement practices
2.4/2.6 Develop, pilot test data-driven quality improvement intervention bundle Evaluate interventions to improve data-driven quality development
2.5 Develop measurement/evaluation concept; test pilot study
2.7 Evaluate data-driven quality development intervention bundle
**Work Package 3: Introducing new national MQIs**
*Objective: To introduce new MQIs in Swiss LTCFs and recommend additional MQIs*
3.1 Review literature to identify internationally used quality indicators, risk adjustment for proposed MQIs
3.2/3.4 Operationalize, introduce three new MQIs
3.5 Evaluate, introduction of the three new MQIs; assess for suitability
3.6 Recommend additional MQIs

LTCF = long-term care facility; MQI = medical quality indicator.

### WP1 – Improvement of Data Quality

The first work package investigates the current quality of the MQI data and its determinants to develop interventions to improve the data quality and strategies for their implementation. Using a participatory approach, the intervention bundle and implementation strategies will be developed, implemented, and evaluated in Swiss LTCFs. Specific objectives of the sub-aims are detailed in Appendix 1.

#### Define Criteria for Data Quality (Sub-Aims 1.1, 1.2)

In a first step, criteria for MQI data quality are developed for the existing MQIs on pain, polypharmacy, physical restraint use, and weight loss, and the proposed new MQIs on pressure ulcers, advance care planning, and medication review. This is the basis for being able to assess and improve the data quality of the MQIs. A common understanding of all steps involved in collecting and preparing high quality data then guides the next steps on how to improve data quality. The literature reviews and workshops with key stakeholders facilitate this process. We expect further insights into important criteria to be considered when assessing current data quality and developing the intervention bundle. Accordingly, a final version of the criteria will be available at the end NIP-Q-UPGRADE.

#### Assess Current Practices and Data Quality (Sub-Aims 1.3, 1.4)

Secondly, an initial contextual analysis (sub-aim 1.3) at all levels of MQI data flow is conducted to explore barriers and facilitators of data quality and data flow processes. The contextual analysis is part of the EPIS preparation phase. It is an important step to understand the determinants of data quality guided by CFIR, for the development of an intervention bundle to improve data quality and to guide the selection of strategies for implementation and scale-up.^26^ Specifically, the methods of collecting and process of transferring MQI data from residents’ clinical assessments to the Federal Offices is investigated with a rapid ethnography approach, including all levels of action (ie, LTCFs (collection and recording of data), EHR software (providing algorithms to simplify data collection, such as calculating weight loss, providing modules to summarize MQI data), resident assessment instruments (summarizing data, making plausibility checks and transfer to Federal Statistical Office) and the Federal Offices (plausibility checks, calculation of the MQIs and public reporting)). We also survey cantons who provide the regulatory context for quality assessment of LTCFs.

Further insights are gained from an interrater reliability study (1.4), assessing the current accuracy of the MQI data and assessing determinants for disagreement in the assessment of resident situations as well as understanding sources of problems for data quality at the level of software providers,. After the implementation of the intervention bundle, this study will be repeated with the same LTCFS to evaluate the impact of the toolbox with the Train-the-Trainer programme (sub-aim 1.9).

#### Analyse and Improve Data Quality Problems at System Level (Sub-Aim 1.5)

In this sub-aim, building upon the feedback and insights from the context analysis in sub-aim 1.3, we liaise and collaborate with stakeholders at multiple levels (Federal offices, software providers) to identify opportunities to further optimize systems to improve data quality. Examples include the uniformization and alignment of algorithms embedded in EHRs, the automatization of the counting of active ingredients used in the calculation of polypharmacy, etc.

#### Develop Measures to Optimize MQI Data Communication (Sub-Aim 1.6)

This sub-aim focuses on developing insights and recommendations for the communication of quality indicator (QI) results to enhance data understanding and support data-driven quality improvement in long-term care for older adults. A 4-step participatory approach was adopted to identify, refine, and implement communication measures with active stakeholder involvement, ensuring that recommendations proposed are relevant and feasible from their perspective.

In the first step, a rapid review ^27^ of the literature (subaim 1.1) is conducted to identify effective communication strategies and methods for improving QI data interpretation and supporting quality improvement efforts.

In the next step, building on insights from the literature review and contextual analysis we conduct workshops with management teams from 4 LTCFs in 2 Swiss language regions to explore their needs and preferences for optimizing QI communication. In a third step, we engage sounding boards and stakeholder groups to collaboratively refine communication strategies and ensure alignment with stakeholder priorities. Finally, we hold workshops and follow-up meetings with software vendors, allocation system representatives, and federal offices to facilitate the practical implementation of communication measures and support ongoing improvements.

#### Develop and Pilot Test Intervention (Sub-Aims 1.7/1.8)

sSub-aims 1.7 and 1.8 serve to prepare the outer as well as the inner context for interventions to improve data quality. Interventions targeting the outer context refer to measures that can be taken outside of the LTCFs, that is, at federal level or the levels of the software providers, to ease the task of reaching good data quality. This can include simplifying the measures (eg, introducing a standardized scale to observe pain intensity) or aligning algorithms between the software providers (eg, how to automatically calculate weight loss). Sub-aim 1.7 serves to reach national agreement on how to improve the measurement between all key stakeholders using workshops, written feedback, and the building of expert sub-groups for single MQI themes to find consensus for specific quality issues.

#### Evaluate Interventions to Improve the Data Quality (Sub-Aim 1.9)

Moving further in the implementation phase of the EPIS framework, 1.9 serves to assess the impact of the measures on the data quality, repeating the interrater reliability study of 1.4. LTCFs that participated in 1.4 are asked to implement the intervention bundle that is adapted following the pilot testing and repeat the routine and gold standard assessment. We will also re-assess the data of the software providers to assess whether the feedback after 1.4 and further measures in 1.5 led to improvements in data quality.

#### Perform Regional Scale-Up and Prepare National Scale-Up (Sub-Aim 1.10)

With sub-aim 1.10, the toolbox with the Train-the-Trainer programme is fully passed over to the national LTCF associations for them to scale it up nationally in all language regions.

### WP2 – Implementing Quality Improvement

The second work package focuses on implementing quality improvements in LTCFs following the EPIS framework as in WP1. Exploration of international evidence and best-practice examples, alongside the exploration of the context in Swiss LTCFs, provide input for the preparation phase involving the development of an intervention bundle using intervention mapping and a participatory approach. The intervention bundle aims to support LTCFs in using their data to inform quality improvement, with a specific focus on the application of the Plan-Do-Check-Act (PDCA) cycle. We will develop a toolbox and training for quality improvement and pilot test it in a convenience sample, aiming to build a scalable unit for national implementation. Specific objectives of the sub-aims are detailed in Appendix 2.

#### Identify Interventions and Implementation Strategies for QI Themes and Supporting Data-Driven Quality Improvement (2.1)

Three literature reviews are performed to guide the development and implementation of a quality improvement programme. An umbrella review following JBI guidelines^28^ focuses on the effectiveness of clinical interventions in QI areas. A rapid review focuses on implementation strategies and key facilitators for data-driven quality development in LTCFs and a second rapid review informs the development of an evaluation concept for the scale-up of a quality development programme.

#### Explore the Literature and International Best Practice Cases in Quality Improvement (Sub-Aim 2.2)

The objective of this sub-aim is to identify and examine processes and best practices in planning, implementing, and sustaining large-scale, data-driven quality improvement strategies in LTCFs for older people in different countries. The reviews (2.1) and international best practice examples in national quality improvement (2.2) serve to develop ideas about how to best foster data-driven quality improvement in the LTFC setting. The review addresses processes and practices of large-scale data-driven quality improvement initiatives, including areas like indicator characteristics, data standards, implementation strategies, reporting, culture change, data collection and transfer. The case studies examine the processes and practices of planning, implementing, and sustaining large-scale, data-driven quality improvement strategies in LTCFs in 3 countries. We select Australia, Canada, and New Zealand as countries with long-running programmes with different approaches to data collection and national reporting.

#### Identify Determinants and Needs of Swiss LTCFs in Quality Development (Sub-Aim 2.3)

Barriers and facilitators of data-driven quality improvement are identified in a mixed-method approach, to guide the development of both intervention and implementation strategies in 2.4. A national online survey with Swiss LTCFs explores current structures, practices, and processes of data-driven quality improvement and identifies gaps and needs in their implementation. In-depth interviews with managers of Swiss LTCFs, quality managers and healthcare professionals further enhance our understanding of the determinants and needs. In addition, 3 workshops with residents and relatives are held to discuss the topic of care quality from their perspective.

#### Develop an Intervention Bundle for Quality Improvement (Sub-Aim 2.4)

Based on the findings from the studies mentioned above, in the preparation phase an intervention bundle for quality improvement will be developed in an intervention mapping process. Stakeholders such as registered nurses, quality leaders and leaders of LTCFs, representatives in sounding boards and national and international experts are invited to participate in the co-creation of the bundle of tools and resources. The performance objectives derived from intervention mapping exercises will guide the selection of interventions and strategies to be implemented, their intended outputs and outcomes, and the processes through which they will achieve their aims. The umbrella review in sub-aim 2.1 will support the development of material around quality improvement by identifying key elements of effective clinical interventions for the existing and new MQIs (cf. WP 3).

#### Develop a Measurement and Evaluation Concept (Sub-Aim 2.5)

The last step of intervention mapping consists of developing an evaluation plan. Based on the results of sub-aim 2.4 and the review of methods for evaluating the intervention bundle for quality improvement and its scale-up, a measurement and evaluation concept is developed that will serve the LTCF associations in evaluating and monitoring the intervention bundle over time. The concept includes assessing implementation and intervention outputs and outcomes, guided by the RE-AIM framework.^29^ Key dimensions such as reach (eg, facility coverage), effectiveness (eg, use of PDCA cycle for quality development), adoption (eg, intention to implement programme components), and fidelity (eg, adherence to protocol, including conducting leadership meetings to communicate MQIs or conducting PDCA cycle steps) are included in this concept.

#### Pilot Test the Intervention Bundle for Data-Driven Quality Development (Sub-Aim 2.6)

The objective of this sub-aim is to implement and test an intervention bundle for data-driven quality improvement, along with a measurement and evaluation framework, across Switzerland’s three main language regions (German-, French-, and Italian-speaking). Building on the EPIS framework and using the Intervention Mapping approach (sub-aim 2.4), a tailored toolbox is developed for internal use in Swiss LTCFs, guided by external training delivered to Quality Leaders nominated within the LTCF and a representative of the LTCF management. The intervention bundle includes a training programme and activities inspired by the insights learned from earlier sub-aims, ensuring the intervention bundle and implementation strategies are context specific. The material addresses activities towards 5 target populations: management, quality leaders, care staff, broader facility staff, residents and relatives.

The pilot test (sub-aim 2.6) involves an initial implementation to evaluate key outcomes such as costs, acceptability, feasibility, and fidelity to the intervention steps.

#### Regionally Test and Scale-Up (Sub-Aim 2.7 and 2.8)

In these last sub-aims, the scale-up of the intervention bundle for quality improvement as well as the measurement and evaluation concept are tested regionally in 3 language regions (2.7). This facilitates assessment of whether the implementation works in a broader context and what modifications are needed before going to full-scale nationally. To assure sustainability, these phases are developed in close collaboration with the LTCF associations so that they can take over the national scale-up and monitoring after NIP-Q-UPGRADE.

### WP3 – Introducing New National Medical Quality Indicators

Work package 3 aims to prepare the field for the introduction of 3 additional MQIs at the national level in Swiss LTCFs. The 3 MQIs proposed are pressure ulcers, medication review, and advanced care planning. The sub-aims include the operationalization of the new MQIs, assessing barriers and facilitators for their implementation, putting in place corresponding implementation strategies, and evaluating their implementation. In addition, a literature review and Delphi study are performed to identify further quality indicators to introduce for a fuller picture of LTCF quality of care. Specific aims are detailed in Appendix 3.

#### Evaluate Further MQIs and Risk Adjustment Variables (Sub-Aim 3.1)

The evaluation of further MQIs for Swiss LTCFs begins with a review of the literature on what quality or life and quality of care frameworks exist internationally, and what quality indicators are in use (sub-aim 3.1). The findings will inform the development of a questionnaire for use in a modified RAND/UCLA study that aims to find consensus on further MQIs. To identify appropriate risk adjustment variables for the new MQIs, a review of the literature will be conducted to assess which variables are adjusted for in international practice (sub-aim 3.1).

#### Define, Operationalize and Introduce the Three New MQIs (Sub-Aims 3.2, 3.3, 3.4)

Consultation with experts in the field will help to operationalize the 3 new MQIs. While the operationalization of pressure ulcer has international examples, special effort is put into the operationalization of ACP and medication review to reach national alignment in the definition and operationalization of these MQIs (sub-aim 3.2). Results from the contextual analysis from work package 1 (sub-aims 1.3 and 3.3) help to guide the operationalization in view of the Swiss context in this preparation phase. In consultation with stakeholders, intervention mapping will guide the development of strategies for implementing the new MQIs in LTCFs’ daily practice (sub-aim 3.4). Intervention mapping will thus also produce information packages to support LTCFs and related stakeholders in the implementation of these processes.

#### Evaluate the New MQIs (Sub Aim 3.5)

The evaluation of the implementation of the new MQIs will address both the processes in place and what changes can be observed before and after the introduction of the measurement, and implementation outcomes (3.5a). The latter include acceptability, feasibility and cost of the measurement. To assess data quality, MQI data retrieved from needs assessment instruments will be analysed to assess whether the new MQIs fulfil statistical requirements of quality indicators (eg, between-provider variability), and can be used for national reporting (3.5b).

#### Recommend Additional Quality Indicators (Sub-Aim 3.6)

The literature reviews conducted in sub-aim 3.1 will provide the foundation for consultations with national and international experts. First, semi-structured interviews with national LTC experts are conducted on the themes emerging from the literature and to identify priority areas. Followed by with a modified RAND/UCLA eDelphi study with both national and international experts, and staff working in LTCFs to assess the importance, feasibility and action-ability of potential additional MQIs. Input from residents and their family members is gathered during workshops and is shared with panel experts in the modified RAND/UCLA eDelphi. The findings will be used to formulate recommendations for further quality indicators in Switzerland.

## Discussion

This overview protocol of NIP-Q-UPGRADE outlines the Swiss context, the guiding frameworks, and the methodologies adopted in this extensive and layered programme. At the conclusion of NIP-Q-UPGRADE, the aim is that the implemented intervention bundles will have strengthened knowledge and processes around quality improvement in LTC, addressed gaps in the research on national MQI data and processes, and fostered constructive dialogue and collaboration across LTCFs, LTC associations, software providers, cantonal and federal offices and academic partners. In the longer-term, the programme’s goal is to build practice capacity with sustainable, long-term impact.

Applying implementation science, NIP-Q-UPGRADE offers practical solutions to support LTCFs in their efforts to develop the quality of care using routine data. It engages with stakeholders at all levels to understand, facilitate and develop the conditions and tools needed for improving the quality of MQI data, using it effectively to inform quality development initiatives and preparing the field for further MQIs. The emphasis on understanding the context, inspired by the guiding frameworks, ensures that the needs and constraints of LTCFs are considered in the development of the intervention bundle. The methods proposed within each work package facilitate bringing together best-practices from the published literature with context-specific needs and conditions to foster the development of tools and strategies that are relevant, acceptable and effective. The participatory approach that involves stakeholders at all levels, including at the facility, cantonal, and national level, to understand the context, develop inter-sectoral solutions, and support LTCFs moving forward lays the foundation scalability and sustainability, as well as for addressing challenges unique to Switzerland, such as the fragmented organisation of care and regional differences

### Strengths and Limitations

The timing of NIP-Q-UPGRADE represents an important strength in terms of its potential impact and sustainability. With the publication of the first reports on the national MQIs in 2023 and 2024,^30,31^ LTCFs are at a phase in their collection and reporting of MQIs where it is integrated in usual practice, but where it is not yet fully consolidated. LTCFs have demonstrated a readiness and motivation to engage with the data and reflect on its quality and its use in their local context for continuous quality development.

A second notable strength of the programme lies in its collaborative approach, bringing together stakeholders across language regions, and fostering regional partnerships and collaborations at multiple levels to understand the context and co-develop solutions and strategies.

This ambitious programme unfolds in a relatively short period of time, potentially limiting the depth with which stakeholders can engage with the development and implementation of the proposed intervention bundles. It also limits the ability to observe longer-term outcomes. Known disparities across Switzerland also pose an important challenge, particularly with respect to differences in facilities’ access to their own data, varying levels of data literacy in the LTCFs, and variation in the user-friendliness of the software packages utilized in the LTCFs.

It is expected that NIP-Q-UPGRADE will offer important insight for similar initiatives in Switzerland and internationally. From a practice perspective, lessons learned will offer insight into how professional training might reinforce the inclusion of MQIs, data quality, and data development into the curricula. From a policy perspective, our findings may have implications for exploring how the maintenance of high-quality data and its use in LTCFs can be supported through regional or cantonal initiatives and monitoring.

## Conclusion

NIP-Q-UPGRADE will contribute to supporting existing, and introducing new, practices in LTCFs, as well as to a relevant policy review to sustainably improve quality of care and continuous quality improvement in Swiss LTCFs. The results will provide data on national quality improvement implementation applicable to a global policy and practice audience. The potential for extending this project to an international context may depend on the existing quality measurement systems. However, it provides the flexibility to determine the starting point for implementation. Logic models for the work packages and/or the individual sub-aim studies make this national programme accessible and adaptable to different contexts. Additionally, open access publications will highlight the various steps of NIP-Q-UPGRADE, providing detailed methodologies to facilitate transferability and replication.

## Supplemental Material

sj-docx-1-inq-10.1177_00469580251328101 – Supplemental material for Strengthening quality of care in partnership with long-term care facilities: Protocol of the Swiss National Implementation Programme NIP-Q-UPGRADE Supplemental material, sj-docx-1-inq-10.1177_00469580251328101 for Strengthening quality of care in partnership with long-term care facilities: Protocol of the Swiss National Implementation Programme NIP-Q-UPGRADE by Nereide A. Curreri, Laurie Corna, Emmanuelle Poncin, Bastiaan Van Grootven, Jianan Huang, Magda Osinska, Serena Sibilio, Lisa Kästner, Simon Thuillard, Lucie Vittoz, Sonja Baumann, Brigitte Benkert, Angelika Rüttimann, Anna Brambilla, Gabriela Cafaro, Nathalie Wellens and Franziska Zúñiga in INQUIRY: The Journal of Health Care Organization, Provision, and Financing

sj-docx-2-inq-10.1177_00469580251328101 – Supplemental material for Strengthening quality of care in partnership with long-term care facilities: Protocol of the Swiss National Implementation Programme NIP-Q-UPGRADE Supplemental material, sj-docx-2-inq-10.1177_00469580251328101 for Strengthening quality of care in partnership with long-term care facilities: Protocol of the Swiss National Implementation Programme NIP-Q-UPGRADE by Nereide A. Curreri, Laurie Corna, Emmanuelle Poncin, Bastiaan Van Grootven, Jianan Huang, Magda Osinska, Serena Sibilio, Lisa Kästner, Simon Thuillard, Lucie Vittoz, Sonja Baumann, Brigitte Benkert, Angelika Rüttimann, Anna Brambilla, Gabriela Cafaro, Nathalie Wellens and Franziska Zúñiga in INQUIRY: The Journal of Health Care Organization, Provision, and Financing

sj-docx-3-inq-10.1177_00469580251328101 – Supplemental material for Strengthening quality of care in partnership with long-term care facilities: Protocol of the Swiss National Implementation Programme NIP-Q-UPGRADE Supplemental material, sj-docx-3-inq-10.1177_00469580251328101 for Strengthening quality of care in partnership with long-term care facilities: Protocol of the Swiss National Implementation Programme NIP-Q-UPGRADE by Nereide A. Curreri, Laurie Corna, Emmanuelle Poncin, Bastiaan Van Grootven, Jianan Huang, Magda Osinska, Serena Sibilio, Lisa Kästner, Simon Thuillard, Lucie Vittoz, Sonja Baumann, Brigitte Benkert, Angelika Rüttimann, Anna Brambilla, Gabriela Cafaro, Nathalie Wellens and Franziska Zúñiga in INQUIRY: The Journal of Health Care Organization, Provision, and Financing
