# Supplementary material for: Strengthening quality of care in partnership with long-term care facilities: Protocol of the Swiss National Implementation Programme NIP-Q-UPGRADE
Source: Inquiry. 2025 May 22;62:00469580251328101. doi: 10.1177/00469580251328101 (PMC12099085; doi:10.1177/00469580251328101)
Supplement: sj-docx-1-inq-10.1177_00469580251328101 – Supplemental material for Strengthening quality of care in partnership with long-term care facilities: Protocol of the Swiss National Implementation Programme NIP-Q-UPGRADE [file sj-docx-1-inq-10.1177_00469580251328101.docx]

**Strengthening Quality of Care and Its Indicators in Partnership with Long-Term Care Facilities**

Appendix 1. Specific objectives of sub-aims in Work Package 1

| **Sub-aim** | **Objective** | **EPIS phase / Scale-up phase** | **Methods used** |
| --- | --- | --- | --- |
| 1.1 | Review and provide an overview of the literature on the determinants of data quality, interventions to enhance data quality, communication strategies to enhance MQI data interpretation, and implementation strategies for scale-up in LTCFs | Exploration | Scoping literature reviews and a rapid review^28^ using databases like Pubmed, Embase, and CINAHL. |
| 1.2 | Define the criteria for the quality of data and related processes | Preparation | Literature overview (including grey literature) to build a first list of criteria. Workshops with stakeholders and written feedback to revise this list. Regular update based on insights in further sub-aims until the end of NIP-Q-UPGRADE. |
| 1.3 | Assess the current practices of data collection and documentation and determinants for MQI data quality (also addresses the three new MQIs introduced in WP3, same content as sub-aim 3.3) | Preparation | Contextual analysis with a rapid ethnography methodology. Sample includes staff of approximately 30 LTCFs in three language regions (interviews, study of documentation, and observations), staff of three needs assessment instruments and approximately 7 EHR providers (interviews, study of documentation, and software demonstration), cantonal representatives (written survey), and representatives from the FOPH and FSO (interviews, study of documentation). Data is analyzed with Rapid Analysis Protocol (RAP) sheets based on CFIR. |
| 1.4 | Validate current MQI data with double assessments and group interviews in LTCFs. | Preparation | 1.4a: Data quality will be assessed at the level of the LTCFs and at the level of the providers of the needs assessment instruments that act as intermediaries in data delivery between LTCFs and the FSO.  LTCFs: Using a multi-method approach, first the interrater reliability will be assessed in 30 LTCFs of three language regions, with the data of 30 residents per facility collected in the usual way and by a trained gold standard assessor. After finishing data collection, focus groups will be held within each LTCF with leadership persons, gold standard and routine assessors to explore determinants of data quality and ideas for interventions to improve data quality. Data will be summarized using RAP sheets based on issues per MQI, and ideas for improvement.  1.4b: Software providers: we compare raw data they receive from the LTCFs with data they pass on to the FSO to assess any uncertainties in the processing of data. |
| 1.5 | Engage stakeholders (Federal Offices, Software Providers) to identify system optimization needs for better data quality. | Preparation / Set-up | Insights from 1.3 will be used to discuss the need for improvement of data quality outside LTCFs and possible measures (e.g., alignment of algorithms used in EHR, automatization of counting active ingredients for polypharmacy, change in quality measures to simplify assessment). |
| 1.6 | Develop measures to optimize MQI data communication, interpretation, and usability. | Preparation / Set-up | The results of the literature review (sub-aim 1.1) and the contextual analysis (subaims 1.3, 2.3) inform workshops with management in four facilities to identify needs for optimizing QI communication. Sounding boards and stakeholder groups shape future communication strategies and are followed by workshops and follow-ups with software vendors, allocation systems, and federal offices to support implementation. |
| **1.7** | Co-develop an intervention package to enhance MQI data quality. | Preparation / Build scalable unit | The Intervention Mapping approach will be used to develop an appropriate intervention bundle that addresses determinants of data quality identified in 1.3. This includes the development of logic models (step 1 and 2); designing an intervention bundle to improve data quality (step 3) considering the ISF mentioned above; producing an intervention bundle with protocols and practical materials adopting a Train-the-trainer model (step 4); defining an implementation plan (step 5); and an evaluation plan (step 6)^25^. |
| **1.8** | Pilot and refine the intervention package in selected LTCFs. | Implementation / Test scale-up | A pilot test is performed with a multi-method evaluation. We recruit 1) educational institutions able to offer the Train-the-Trainer program developed in 1.7 in three language regions, providing teachers with the materials needed to offer the training, 2) a convenience sample of 15-30 LTCFs (5-10 per language region) to complete the training and implement the intervention bundle locally by introducing Champions to foster data quality. We assess implementation outcomes such as acceptability, feasibility, fidelity and costs at all levels with semi-structured interviews (Trainers) and focus groups (LTCF leadership, Champions), an online survey (LTCF staff) and activity sheets (teacher, LTCF leadership). We use a rapid qualitative analysis approach for qualitative data and descriptive analysis for quantitative data. |
| **1.9** | Evaluate the impact of the intervention package on MQI data quality. | Implementation / Test scale-up | The study design of 1.4 is repeated at both LTCF (1.9a) and software provider level (1.9b) to assess differences in data quality. |
| **1.10** | Disseminate the intervention package to LTCFs. | Implementation and Sustainment / Got to full scale | Based on the former sub-aims, the research team will guide the national LTCF associations with recommendations concerning scale-up strategies in the further national implementation of the developed toolbox (or intervention bundle) with the Train-the-Trainer programme and practical materials as well as with a concept for monitoring and evaluating the scale-up. |

Abbreviations: EHR electronic health records, FOPH Federal Office of Public Health, FSO Federal Statistical Office, LTCF long-term care facility, MQI medical quality indicator
