# Supplementary material for: Strengthening quality of care in partnership with long-term care facilities: Protocol of the Swiss National Implementation Programme NIP-Q-UPGRADE
Source: Inquiry. 2025 May 22;62:00469580251328101. doi: 10.1177/00469580251328101 (PMC12099085; doi:10.1177/00469580251328101)
Supplement: sj-docx-2-inq-10.1177_00469580251328101 – Supplemental material for Strengthening quality of care in partnership with long-term care facilities: Protocol of the Swiss National Implementation Programme NIP-Q-UPGRADE [file sj-docx-2-inq-10.1177_00469580251328101.docx]

**Strengthening Quality of Care and Its Indicators in Partnership with Long-Term Care Facilities**

Appendix 2. Specific objectives of sub-aims in Work Package 2

| **Sub-aim** | **Objective** | **EPIS phase** | **Methods used** |
| --- | --- | --- | --- |
| **2.1** | Review the literature on 1) interventions to improve MQI themes, 2) interventions to foster data-driven quality improvement, and 3) ways of evaluating scale- up programmes | Exploration | Three literature reviews are performed using databases like Medline, Embase, CINAHL, PsycInfo or Cochrane Library: (1) an umbrella review following JBI guidelines^30^ (2) a rapid review; and (3) a methods review. |
| **2.2** | Identify and derive lessons learned from international data-driven policy programmes in quality improvement. | Exploration | A collective case study approach is used. It is informed by scientific and grey literature and supplemented by five semi-structured interviews with eight experts actively involved in nationwide quality programs in three countries (Australia, Canada, and New Zealand). |
| **2.3** | Assess the current quality improvement practices in LTCFs. | Exploration / set-up | An explanatory-sequential quantitative-qualitative mixed-method design is used. In the first quantitative step, a national online survey of the full sample of all LTCFs in Switzerland (n=approx. 1485) is performed to identify current structures, practices and processes for care quality improvement with special focus on the current MQIs. Participants are asked about their willingness to participate in a follow-up interview for an in-depth understanding of quality improvement practices. Of those interested, a purposeful, heterogeneous sample of 15 LTCFs from all three language regions is selected for a semi-structured interview.  In addition, 4 workshops with approximately 12 residents and 12 family members are held to explore their perspectives on quality of care and quality improvement. |
| **2.4** | Develop an intervention bundle for quality improvement and associated implementation strategies. | Preparation / Build scalable unit | As in 1.7, an intervention mapping approach is used to develop an appropriate intervention bundle that addresses determinants of data-driven quality improvement. This includes the development of performance objectives for target populations (step 1 and 2); designing an intervention bundle to support data-driven quality development (step 3); producing an intervention bundle with tutorials and practical materials for training and application of activities within the facility (step 4); defining an implementation plan (step 5); and an evaluation plan (step 6). |
| **2.5** | Develop an evaluation concept to assess the outcomes of the intervention bundle. | Preparation / Build scalable unit | Based on the results from the methods review (2.1 – 3) and in collaboration with the national LTCF associations, an evaluation concept is developed to assess the program outputs and outcomes developed in 2.4. |
| **2.6** | Pilot test an intervention bundle for data-driven quality improvement | Implementation / Test scale-up | The same LTCFs enrolled in 1.8 are involved in the second phase of the pilot, testing the intervention package developed in 2.4. We recruit educational institutions able to offer the trainings developed in 2.4 in three language regions. Quality Leaders in the care homes, alongside a colleague from management, are provided with the materials needed to implement the measures locally. We assess implementation outcomes such as acceptability, feasibility, fidelity and costs at all levels with semi-structured interviews (teachers) and focus groups (care home management, quality leaders), online survey (LTCF staff) and activity sheets (teacher, LTCF management). We use a rapid qualitative analysis approach for qualitative data and descriptive analysis for quantitative data. |
| **2.7** | Regionally test a measurement and evaluation concept for a national evaluation of the intervention bundle for data-driven quality improvement, assessing outputs and outcomes. | Sustainment / Test scale-up | We assess the output and outcome of the training in a regional test of the scale-up with one region (e.g., canton) in each language region. Trainings will be offered by different educational institutions. We recruit up to 30 LTCFs participating in the training from selected cantons to assess, for example, the degree of implementation of intervention elements in the LTCF and the determinants of implementation success or failure using online surveys and in-depth, semi-structured interviews with a sub-sample of LTCF leadership and care professionals. We also select a sub-sample of LTCFs residents for a qualitative interview to assess the impact of the intervention bundle and Train-the-Trainer programme at the resident level. We assess the reach of the Train-the-Trainer programme via the educational institutions. |
| **2.8** | Disseminate the intervention package to LTCFs. | Implementation / Go to full scale | As in 1.10, the research team will guide the national LTCF associations in scaling-up the intervention bundle to full scale and support its monitoring. |
