# Supplementary material for: Strengthening quality of care in partnership with long-term care facilities: Protocol of the Swiss National Implementation Programme NIP-Q-UPGRADE
Source: Inquiry. 2025 May 22;62:00469580251328101. doi: 10.1177/00469580251328101 (PMC12099085; doi:10.1177/00469580251328101)
Supplement: sj-docx-3-inq-10.1177_00469580251328101 – Supplemental material for Strengthening quality of care in partnership with long-term care facilities: Protocol of the Swiss National Implementation Programme NIP-Q-UPGRADE [file sj-docx-3-inq-10.1177_00469580251328101.docx]

**Strengthening Quality of Care and Its Indicators in Partnership with Long-Term Care Facilities**

Appendix 3. Specific objectives of sub-aims in Work Package 3

| **Sub-aim** | **Objective** | **EPIS phase** | **Methods used** |
| --- | --- | --- | --- |
| **3.1** | Review the literature to 1) identify quality indicators used internationally that could be considered for Swiss LTCF and 2) identify risk adjustment variables for new MQIs | Exploration | Three rapid reviews are performed to gain a broad understanding of quality of care and quality of life frameworks to subsequently assess internationally used quality indicators, including patient-reported outcome and experience measures. For the second objective, a scoping review on variables for risk adjustment is carried out to prepare for risk adjustment in public reporting for the new MQIs. |
| **3.2** | Operationalize the three new MQIs: pressure ulcers, advanced care planning (ACP), and medication review, and prepare their implementation. | Preparation | Adopting a participatory approach with stakeholders from the Federal Offices, needs assessment instruments, electronic health records providers, professional organizations, and experts from the practice setting, all three MQI are operationalized. For ACP, close collaboration with an existing national working group mandated by the Federal Office of Public Health to promote ACP in Switzerland is sought to align efforts. For medication review, expert interviews are followed by a RAND/UCLA eDelphi study to have a national understanding of the criteria for conducting a medication review in LTCFs. |
| **3.3** | Assess the context for implementing the new MQIs (same as 1.3) | Preparation | Same as 1.3 |
| **3.4** | Develop measures and corresponding implementation strategies to support the introduction of the new MQIs. | Preparation | As in 1.7 and 2.4, an intervention mapping approach is used to identify intervention elements needed to implement the new MQI, especially the processes needed to perform ACP and medication reviews, and the corresponding implementation strategies to foster their use and the measurement of the MQIs. |
| **3.5** | Evaluate the implementation of the new MQIs | Implementation | 3.5a: A mixed-method design is adopted to evaluate the implementation of the new MQIs with a pre-post study. Both before and after the introduction of the new MQIs, a quantitative national online survey of LTCF leaders is fielded to assess current structures and processes regarding the MQIs and implementation outcomes such as feasibility and adoption. Within this one group pre-post design, intra-LTCF comparison will be made between the LTCFs that participate in both waves of surveys, as well as pooled comparison between the two waves.  Post-implementation, additional qualitative interviews are conducted to explore in-depth experiences, perceptions, and expectations with the new MQIs. Thematic analysis is used to capture the challenges encountered during introduction of the new MQIs and the related processes.  3.5b: In addition, the data of the new MQIs are evaluated to assess their quality, including extent of missing data, between-provider variability and rankability. Data will be exported via the needs assessment instruments from LTCFs that already introduced the new instruments needed to assess the MQIs. |
| **3.6** | Identify additional quality indicators through best practices and stakeholder consultations. | Exploration | Using a modified RAND/UCLA e-Delphi panel method, we first conduct semi-structured interviews with national experts to get their feedback on the findings of the literature reviews (sub-aim 3.1). The findings inform the development of the questionnaire used in the eDelphi with national and international experts. Experts rate each included indicator according to its importance, feasibility and actionability in the Swiss LTC context. Between the first and second round, an online workshop will be held with panel members to: a) present the findings of the first round and discuss discordant results; b) share the findings that emerge from workshops on further quality indicators to be held with LTCF residents and their family members in each of the four Regional Health Director’s conferences.  A second and final round of rating with the panel will be completed following the online workshop. |
